# Intelligence-led policing in the 21^st^ Century: How increased mobility requires new paradigms of information sharing

**DOI:** 10.1177/0032258X241309479

**Published:** 2024-12-20

**Authors:** Rebecca Phythian, Stuart Kirby

**Affiliations:** School of Law and Criminal Justice, 6249 Edge Hill University, Ormskirk, UK; School of Law and Policing, 6723 University of Central Lancashire, Preston, UK

**Keywords:** Foreign national suspects, mobile criminality, information sharing, law enforcement, transnational serious organised crime, mobility, intelligence-led policing

## Abstract

The challenge posed by mobile criminality to law enforcement has increased in the 21^st^ Century, as technology and digital communication have accelerated. This study examines the threat by analysing foreign national suspects data (arrested in a UK police force, n = 293) and UK-based practitioner interviews (n = 36). The evidence reveals the threat from offenders who travel between countries is growing, in quantity and sophistication. To keep pace with this evolution, law enforcement must develop new paradigms of information sharing, using technological advances and machine learning to their benefit and relying less on resource-intensive human practice. It suggests such a change will create cultural challenges.

## Introduction

For law enforcement agencies to remain relevant, effective, and efficient they must keep pace with societal change. Whilst the physical mobility of people, commodities, and information, has always generated challenges for law enforcement agencies, this task has become more acute in the 21^st^ Century. Technology and digitisation have underpinned a proliferation of online mobility, which has transformed the criminal landscape. Whilst the National Intelligence Model (NIM) emerged at the start of the millennium with a framework to tackle mobile criminality, this paper questions whether it remains sufficiently agile to frustrate the criminogenic opportunities of a developing 21^st^ Century. Specifically, it argues new paradigms are needed in relation to information sharing across law enforcement agencies.

### The relevance of mobility to policing

It has been argued law enforcement agencies find themselves in an arms race with offenders, as each constantly try to establish an advantage over the other (Ekblom, 2003). One of the factors affecting this has been mobility, which has influenced policing approaches throughout history. The United Kingdom (UK) epitomises this struggle. The urbanisation of the industrial revolution became a catalyst for the creation of the ‘new police’ in 1829, to tackle the unruliness of factory workers who flocked into London to take advantage of higher wages (Mayhew, 2005). Indeed, the instigation of the Metropolitan Police detective branch in 1842 was an early response to mirror the flexibility and mobility of their suspects (Rawlings, 1999, 2002). Mayhew (2005: 103) comments on the “…importation of girls into London from foreign countries to swell the ranks of prostitution in the 19^th^ Century”. As Rawlings (1999, 2002) has highlighted, local police jurisdictional boundaries have continually been exploited by career offenders. Concern in the UK about these issues go as far back as 1962 when a Royal Commission criticised the reticence of police to collaborate with their neighbouring force or introduce modern methods to tackle ‘travelling criminals’ (Willink, 1962). Whilst the Commission argued for a national force or larger regional forces to tackle the problem (Langley, 1965), the ensuing response was less radical. A trickle of innovations, including radios, motorised radio patrol, and fingerprint identification were introduced to keep pace with mobility and although 1974 brought an organisational restructure, there remained 43 territorial police forces in England and Wales, which maintained the focus on local policing (Kirby, 2013).

A tipping point emerged with the onset of ‘late modernity’ during the 1970s, which allowed citizens (including offenders) to travel further, at less cost. It was also associated with a disproportionate rise in reported crime and requests for police service. The traditional policing model – where officers would attend and deal with incidents in isolation – was no longer considered fit for purpose (Kirby, 2013). There was a strong consensus that the police should become intelligence-led, targeting their resources at persistent offenders and repeat problems. UK police leaders commissioned the Baumber, Pearce and Ratcliffe reports (Association of Chief Police Officers [ACPO], 1975, 1978, 1986) to establish how this approach should look in practice. Central to the plans was that each person of interest would be assigned a unique intelligence record, accessible to each police force. Unfortunately, this was not implemented, and each law enforcement agency introduced its own intelligence system, which subsequently generated duplicate records, diverse approaches, and questionable standards (Audit Commission, 1993).

To generate uniformity of approach, the UK NIM was introduced in 2000 with 816 pages of guidance (National Crime Intelligence Service, 2000). A core element of the model was that mobility should be divided into three levels, allowing more effective management of mobile offenders. Level 1 offenders were those who remained in a local policing area. Level 2 offenders were those who crossed local and force boundaries, or who committed offences at a regional level. Level 3 related to those who offended at a national and international level. This conceptualisation allowed resources to be apportioned to each of the tiers, thereby, *in theory,* ameliorating the duplication of effort and allowing the seamless movement of intelligence. The model was embraced internationally, having been implemented in Australasia and North America, as well as parts of Europe and the UAE (Brown, 2007; Kirby, 2013; Ratcliffe, 2006). In 2003 the United States (US) Government endorsed the National Criminal Intelligence Sharing Plan (US Department of Justice, 2003), and a year later the Hague Programme within the European Union officially declared ‘intelligence-led policing’ within its five-year strategy (Council of the European Union, 2005: 22).

### Mobility in the 21^st^ Century

Arguably the 21^st^ Century has presented another tipping point. Epitomised by a networked, market-driven world, historical borders have become less relevant (Kesselring and Vogl, 2004). At a physical level, people and commodities can be transported faster and less expensively than ever before, either by: sea, from container shipments to homemade submarines; land, utilising lorries, cars and human couriers; or air, through postal service, air freight, non-commercial light aircraft and human mules. However, this physical movement has been expanded with the increased use of the internet, supported by social and digital media (Nguyen, 2021). Indeed the 21^st^ Century has introduced a fusion between the physical and online world, leading to a more interconnected and interdependent space (Schwab, 2015). As Lück et al. (2006) explain, bridging distance and overcoming borders presents opportunities for new social interactions, which also benefits offenders. This was illustrated by Tudor (2024), who reported that 12% of the prison population in England and Wales at the end of 2023 were foreign nationals, with UK police forces commonly reporting on the arrest of foreign national suspects (FNS) (e.g. Metropolitan Police Service, 2022; Police Service of Northern Ireland, 2023; West Yorkshire Police, 2021). Unfortunately, these studies do not provide further information as to patterns of offending.

This recent online mobility has transformed the criminal landscape. Traditional crimes, such as theft and burglary, have reduced, ostensibly being replaced by internet-facilitated crimes involving fraud and cybercrime. Such crimes account for approximately half of all crime in the UK (Andrews et al., 2018; Office for National Statistics, 2022; The Police Foundation, 2022). Online opportunities have particularly assisted criminals, who frequently use technology to operate across international and digital borders (National Crime Agency [NCA], 2021), allowing them, “to commit crimes and hide their gains… and exploit vulnerable people with complex needs” (The Police Foundation, 2022: 10). Globalisation has created flexible markets, which generate commercial opportunity. These changes evolve at a faster pace than state structures (Urry, 2007). As such, changes in mobility have the potential to benefit criminals who can adapt quickly. Unfortunately, this is less beneficial to law enforcement agencies, who remain constrained by national structures and protocols (Kirby and Penna, 2011). This is no longer a peripheral issue. Economic crime is of particular concern, so too is online child sexual abuse, with an estimated 850,000 individuals in the UK using the internet to engage with children or remotely view indecent images (NCA, 2021). A rise in organised crime and transnational offending is also identified worldwide (Australian Federal Police, 2017; Gelles et al., 2020; National Police Chiefs’ Council, 2020; Young and Meli, 2019). Indeed, the UK report 4772 organised crime groups (OCGs) comprising 70,000 offenders (NCA, 2021).

The question therefore arises as to whether law enforcement can keep pace with the information needed to identify and track criminality. Legislation, operating procedures, and organisational culture all serve to impede the sharing of information by police forces (Kirby and Penna, 2011). This raises the question whether current intelligence-led approaches, implemented in many developed countries, are sufficiently agile to cope with the diversity and volume of crimes committed across physical and virtual space. As the Council of Europe (2004: 12) argued, “Almost any study on organised crime will point to the fact that the level of cross border co-operation between law-enforcement authorities is trailing far behind the efficiency by which organised crime groups operate transnationally”.

### Hope for the future?

Nonetheless, a few factors provide some room for optimism. First, research shows human and criminal behaviour has a level of predictability, which would allow offenders to be tracked. Brantingham and Brantingham (1995) were amongst the first to show routine geographical patterns in crime, and this continues to be relevant. Song et al. (2010) have shown individuals exhibit 93% predictability in their movements, whether they favour long (over 100 miles) or short journeys. This human predictability has also been observed in social interaction and shopping preferences (Krumme et al., 2013; Zhang et al., 2021). A second investigative benefit is aligned with the fact that offenders find it difficult to engage in activity without leaving an electronic trace. Physical movement, communication, and financial purchases are all captured through a range of electronic surveillance introduced for a diversity of business and safety reasons, including the prevention of crime (Caputo, 2014). High profile reactive criminal investigations have shown how capturing these electronic traces can be pieced together to suggest the guilt of the offender (Kirby, 2013). For proactive investigations (i.e., arresting serial offenders in the planning or commission of crime), the challenge is harder as the relevant information must be identified from voluminous data sources (Denley and Ariel, 2019). This requires adeptness at information management.

In summary, this study examines whether more effective and efficient information management is required across law enforcement agencies, using two independent and distinct samples of data. First, it examines individuals who have travelled to the UK and have been arrested on suspicion of committing crime by examining a sample of FNS arrested by a UK police force. Secondly, it explores how law enforcement practitioners view the challenge created by offenders who transcend borders, examining whether current methods of intelligence sharing remain efficient and effective.

## Method

Identifying mobile offenders is not a simple task. There is no common definition for mobile or transnational offenders or crimes in the academic literature (Von Lampe, 2021). Further, police systems do not record the level of offender mobility. However, police in the UK identify and record a FNS as “a person known or suspected to be involved in criminality who cannot be confirmed as a British citizen at birth” (Sussex Police, 2022). The designation is important as it requires contact with other jurisdictions (and/or checking international databases), to confirm identity and immigration status, as well as review their offending history (College of Policing, 2022).

The study used a mixed methods approach. The first stage examined secondary quantitative data: a detailed sample of 293 foreign nationals, arrested within a UK police force, over a three-month period (between 1^st^ April and 30^th^ June 2022). As well as demographic details (i.e. sex, nationality), this dataset included information about the number of visits to custody (i.e. the number of arrests) and the reason(s) for the arrest (i.e. the type of offence, e.g. theft, public order). Whilst the police organisation asked to remain anonymous, the sample is taken from a mid-sized UK police force, responsible for the policing of both town and rural areas. There is nothing in relation to its geographic or demographic profile which make it more likely to attract foreign nationals; in fact, as a location devoid of a seaport or airport it could be expected to attract less. Some further caveats are also pertinent. It should also be recognised the data reflects arrests in one local force area over a short time period; it is possible that other arrests were made in this force area, by a regional or national crime unit (i.e. Regional Organised Crime Unit [ROCU], NCA) and conveyed to another force area, although this would constitute a small number. Finally, when discussing FNS it is important not to conflate other terms. For example, FNS data does not describe the level of mobility prior to the offence, nor does it identify whether the offender is or has been involved in organised crime during the period spent in the UK.

The quantitative data was subjected to descriptive analysis using SPSS software. This type of analysis provides simple and factual evidence to illustrate information and trends, being less influenced by researcher bias (O’Leary, 2004). However, the weakness of police data generally is that it only describes a proportion of offenders, or suspects, and can be more of a reflection of police effectiveness in tackling a particular phenomenon (Jupp, 1989). In summary, the sample offers a snapshot into the type and scope of offences suspected to be committed by foreign nationals who have travelled to the UK.

The second stage collected qualitative data in the form of practitioner views concerning the level of mobile criminality and the efficacy of intelligence-led approaches when tackling them. Qualitative methods help explain why a certain behaviour occurs and provides meaning (Sarantakos, 2005). In this study, 36 participants engaged in semi-structured interviews, conducted either in person or online (Microsoft Teams). Participants were identified using purposive and snowball sampling techniques and all were in current, or recent, intelligence-related roles. The interviewees comprised a diverse group, with roles ranging between Chief Police Officer and Director level posts to intelligence officers and analysts. The participants were linked to a range of organisations, including the police, NCA, HM Revenue and Customs (HMRC), Border Force and National Trading Standards, as well as non-governmental organisations (NGOs) and the commercial sector (international technology company, animal welfare groups). The respondents were asked, “What do you feel are the challenges that mobile/cross border offenders generate in terms of law enforcement?”. This was followed by a prompt to inquire whether the challenges had changed over the past decade, and if law enforcement were equipped to combat such crimes. They were then asked to comment upon future challenges and opportunities which could either frustrate or improve the sharing of intelligence. The study followed appropriate ethical procedures, and all respondents provided informed consent before taking part (Punch, 1986). Braun and Clarke’s (2006) six-stage method was adopted to thematically analyse the qualitative data, using NVivo software.

## Results

The first stage of the study provides information concerning 293 FNS who were arrested a total of 321 times by the donor force during a three-month period. These comprised 8% of all suspects arrested during this period. Suspects were aged between 13 and 77 years (M = 35.00, SD = 11.30) with males accounting for 84.0% (n = 246) and females 16.0% (n = 47). Nationality was spread across 63 countries, with Europe the most common continent, followed by Asia and Africa. Antarctica was the only continent not represented (see Table 1).

**Table 1.** FNS nationality.

Nationality	% (n)	Nationality (*Country of Nationality*)	% (n)
European	69.3 (203)	Eastern European (*Bulgaria, Czech Republic, Hungary, Moldova, Poland, Romania, Russia, Slovakia, Ukraine*)	43.0 (126)
		Northern European (*Channel Islands, Irish Republic, Latvia, Lithuania*)	13.0 (38)
		Southern European (*Albania, Cyprus, Greece, Italy, Portugal, Slovenia, Spain, Turkey)*	11.3 (33)
		Western European (*Belgium, France, Germany, Netherlands*)	2.0 (6)
Asian	14.7 (43)	South Asian (*Bangladesh, India, Nepal, Pakistan, Sri Lanka*)	7.5 (22)
		West Asian and Middle Eastern (*Iran, Iraq, Israel, Kuwait, Syria*)	2.4 (7)
		East Asian (*China [People’s Republic of], Hong Kong)*	2.0 (6)
		Central Asian (*Afghanistan*)	1.4 (4)
		Southeast Asian (*Malaysia, Philippines*)	1.4 (4)
African	11.6 (34)	East African (*Eritrea, Kenya, Malawi, Mauritius, Somalia, Zimbabwe*)	4.8 (14)
		West African (*Gambia, Ghana, Guinea, Nigeria*)	3.8 (11)
		South African (*Botswana, Namibia, South Africa*)	1.7 (5)
		North African (*Egypt, Sudan, Tunisia*)	1.0 (3)
		Central African (*Congo*)	0.3 (1)
North American	2.4 (7)	North American (*Canada, Jamaica, Mexico, United States*)	2.4 (7)
South American	1.4 (4)	South American (*Brazil, Trinidad and Tobago*)	1.4 (4)
Australian	0.7 (2)	Australian (*Australia*)	0.7 (2)

Across the 321 arrests, a total of 471 arrest reasons – or offences – were recorded. The majority of arrests were for one crime (67.3%, n = 216) with one arrest featuring 10 offences (M = 1.47, SD = 0.88, range = 1-10). Nearly a third of all arrest reasons related to violence against the person, followed by sexual and driving offences. Dishonesty offences, such as theft, fraud or burglary were much lower in frequency. Similarly, online offences, including the passing of indecent images, were also observed, albeit in small numbers (see Table 2).

**Table 2.** Arrest reason according to offence type.

Arrest reason (offence type)	% (n)
Violence against the person (*ABH, assault by beating [incl. of emergency worker], common assault, GBH, attempted murder, death by dangerous driving, false imprisonment, harassment, kidnapping, stalking, threats to kill*)	29.5 (139)
Sexual (*attempted rape, exposure, incite a child, rape [child 13/14/15, female 16 or over], sexual assault [child under 13, child 13 to 15, female over 16, male over 16], voyeurism*)	12.7 (60)
Driving (*disqualified driving, drive motor vehicle without insurance, drive other than in accordance with a license, drive whilst unit drink/drugs, drunk in charge of motor vehicle, fail to provide a breath test, positive breath test*)	11.9 (56)
Other (*admin warrant/miscellaneous, breach of the peace, other, warrant – failed to appear, warrant – no bail, warrant – other, wasting police time*)	10.6 (50)
Public order (*affray, breach of criminal behaviour order, breach of domestic violence prevention order, breach of harassment order, breach of non-molestation order, drunk and disorderly, outraging public decency, racial/religious/hate public order, S.4/4a/5 Public Order Act 1986*)	8.7 (41)
Arson and criminal damage (*arson, criminal damage [under £5000, over £5000]*)	6.4 (30)
Theft (*Blackmail, fraud, general theft inc. shoplifting*)	5.7 (27)
Drugs (*concerned in the supply of controlled drug, cultivation of cannabis, possession of controlled drug [class A, class B], possession with intent to supply controlled drug [class A,B]*	4.9 (23)
Miscellaneous crimes against society (*Communications Act 2003, going equipped, immigration offences, make indecent images, obstruct police, recall to prison*)	3.6 (17)
Possession of weapons (*possess an imitation firearm, possess firearm, possess pointed/bladed article, possession of an offensive weapon*)	3.4 (16)
Burglary (*burglary – dwelling*)	1.3 (6)
Vehicle (*theft – from motor vehicle, theft – of motor vehicle, taken without owner’s consent*)	0.8 (4)
Robbery (*robbery*)	0.4 (2)

Whilst most were arrested once, 24 (8.2%) of the FNS were arrested on up to three occasions and accounted for 52 arrests (M = 2.00, SD = 0.38, range = 2-3) involving 80 offences (M = 3.33, SD = 1.20, range = 2-6). There was an average of 18.42 days between the first and second arrest (SD = 16.00, range = 0-63, n = 24), and 22.75 days between the second and third arrest (SD = 24.55, range = 1-45, n = 4). All but one of the suspects were male, with an average age of 36.04 years (SD = 7.77, range = 20-46 years). Most repeat offenders were European (79.2%, n = 19), followed by African (8.3%, n = 2) and Asian (8.3%, n = 2), with one North American (4.2%). At the time of their first arrest, 54.2% (n = 13) stated they were unemployed.

The second part of the study analysed the interview data collected from 36 practitioners. As these practitioners worked within, or alongside, organisations with a national responsibility their responses often emphasised the role of mobility in organised crime. Specifically, five themes emerged: i) increase in mobile offenders, ii) creativity and adaptivity of offenders, iii) avoiding police attention, iv) combatting mobile criminality and v) improving intelligence sharing. These will be explained in greater detail.

### Increase in mobile offenders

There was unanimous agreement that the threat posed by mobile offenders, specifically transnational organised criminals, had increased within the UK, with one participant estimating that “*90% of the serious organised crime jobs… have got some international element to them”* (P10). Due to their high numbers, it appeared transnational offenders were no longer a niche issue for local police forces:

*“yeah, it has definitely changed, and the world is getting smaller… an OCG would have got their drugs from Manchester, they're now actually going straight to someone in Colombia because they're able to make contact with them. It's such an easy thing to do now, isn't it? So as a consequence, I think our life becomes a bit more difficult and theirs probably becomes a bit more easier”* (P10).

*“about 12-13% of our arrests are foreign nationals, around 12-13% of our prison population, but about 30% of our membership of our map to OCG are foreign nationals”* (P12).

*“what I'm seeing daily now is that there is less and less and less proportion of the crimes that come through that don't have international inquiries”* (P15).

*“I think we've seen a real step change in the last 20 years from people who were just basically cross-border offenders and that was mainly contained within a region, to transnational organised crime”* (P35).

### Creativity and adaptivity of offenders

Practitioners felt offenders had become more creative and adaptive in their ability to commit crime. A common term used by participants was poly-criminality, to indicate that organised crime offenders were constantly exploiting new criminal pathways. P4 highlighted some offenders had responded to new consumer demands in terms of illicit drugs. The officer explained synthetic opioids (e.g. fentanyl), as well as cannabis edibles (sold in the form of confectionary) were becoming increasingly popular. P4 also explained that offenders were constantly adapting their techniques. The use of technology was increasingly common (i.e. the dark web and encrypted communication) as well as community-based courier lockers (which had been introduced to service the increase in online sales, i.e. eBay, Amazon). This evolution allowed offenders to maximise their profits whilst also allowing themselves and their recipients to remain anonymous.

Several participants used the Covid pandemic as an illustration of offender creativity and adaptability. It was explained that the government offer of financial business support was immediately exploited by transnational criminal gangs who provided fictitious company details for support loans and furlough payments. Much of this criminality was conducted electronically allowing the offender to live significant distances from where the crime takes place. P27 also explained the ingenuity of offenders to remain physically mobile even at the height of the covid lockdown, *“they’re there, getting the uniforms for the people who are allowed to travel, getting their friends or associates to get false documentation to allow them to travel freely so they could carry on doing their criminality*. *It takes us years to have that level of sophistication within the police, we’re so far behind*. *It’s just ridiculous really”*.

### Avoiding police attention

Other participants disclosed that offenders would explicitly use their mobility to avoid police attention. P27 said, *“So organised crime groups are very aware that if they commit offences across borders*, *quite often those police forces won’t talk to each other, so they won’t pick up on it*. *They can travel freely and do two offences in one county, move on to the next county and it’s a completely different police force”*. The practitioner disclosed that these offenders would be very selective in terms of a particular crime where a future buyer was known. For example, vehicles would be stolen to order (including agricultural plant) and transportation would be instigated prior to the offender fleeing the country. Participants also explained efforts were made to exploit police procedure. Due to the demand, police proactive teams have thresholds which must be met before they are assigned to investigate an OCG. Offenders are aware of this and often adapt their behaviour to remain under these thresholds. P28 provided an example related to a Roma gang, who were tracked across the UK, Europe, and the USA. The offenders would target exclusive jewellers and either steal luxury watches or dupe staff into refunding money that was not owed to them. The prolific nature of these offenders meant the culmination of such crimes amounted to £millions over the course of the year. However, as the offences involved non-violent shoplifting or fraud, and often reported and recorded as individual crimes, they were not viewed as a priority and not selected for pro-active action.

### Combatting mobile criminality

Participants were then asked to comment on how they felt law enforcement agencies were able to combat this growing threat created by mobile criminality and consensus converged around three points. First, as previously highlighted, the ability to track mobile criminals is made more problematic due to a fragmented approach to information sharing. Participants explained numerous agencies were often involved in proactive investigations, and these appear to be growing. For example, units cited in the UK included the NCA and ROCUs, as well as those government agencies with an intelligence and investigative responsibility (i.e. HMRC, Border Force, Gangmasters and Labour Abuse Authority [GLAA], Department of Work and Pensions, Serious Fraud Office, National Fraud Intelligence Bureau, and ACRO Criminal Records Office). These are supplemented by a variety of NGOs and private companies who also tackle SOC including the Civil Aviation Authority, numerous animal welfare groups (i.e. Royal Society for the Prevention of Cruelty to Animals [RSPCA]) and those targeting piracy and counterfeiting of property (i.e. Federation Against Copyright Theft [FACT]). Practitioners pointed out these organisations used individual intelligence systems which were incompatible and difficult to access. This fragmentation was further complicated by the addition of dedicated national databases (i.e. violent offenders, missing people and organised exploitation). Practitioners also added that intelligence sharing is exacerbated when the information is held in another county. This is because investigators are often required to know the specific information exists before an application is made to access it. The dissemination process often involves legislation and protocols tailored to the country which are generally complex and bureaucratic. Further, they often require written rather than digital application, reducing the speed of dissemination (P21).

Moreover, practitioners highlighted the difficulty law enforcement agencies experienced in keeping pace. P12 viewed it from the perspective of the OCG: *“I decide today (because the police are using tactics to stop my high value car burglaries) [to] move that bit to Spain and I can move my pop-up brothels from Spain to the UK because today it’s more lucrative. I can move my cocaine supply to focus on methods in France and get more money… I can bring in teams who will walk into supermarkets [and] fill up trolleys with alcohol completely unchallenged and make me phenomenal amounts of money, and I can ship that to the UK tomorrow. You know, organised crime is highly adaptable”*. P27 emphasised the same point, *“the criminals evolve quickly, don’t they? And we don’t. By the time we’ve changed the legislation, they’ve moved on to something else*. *I know we’re never going to be able to get away from the systems that are currently in place with regards to legislation because that’s the way democracy is and the way that we work with it at the moment, but there’s got to be a way where we can learn to be quicker and there’s got to be a way where policing can evolve quicker”*. P26 also explained that whilst the police have specialist capabilities these are often uncovered by offenders and circumvented.

### Improving intelligence sharing

Faced with these challenges, participants were asked how intelligence sharing could be improved to increase effectiveness and efficiency of tackling offenders who cross borders. The most cited solution was for individual police forces to work off a single system, to aid compatibility. Participants thought this could be possible if there was a commitment to migrate to a common system, with different forces joining when their legacy system expired. It was pointed out that the commercial sector was increasingly providing tools to use single search terms over diverse databases. P21, who was from the commercial sector, argued this approach was feasible for several reasons. First, the technology is available; second, different authority levels could be set for different levels of access; third, people can be vetted prior to being given access; finally, there is accountability built into the system as an audit trail allows all people using it to be registered. The Police National Database (PND) was cited as the only database that provides access between various systems. It allows a single issue to be explored over a variety of otherwise unconnected intelligence databases.

## Discussion

Mobility has created opportunities for business, leisure, and crime. Since the mid 19^th^ Century, the UK has documented the challenges posed to law enforcement by travelling criminals. This challenge was amplified in the 1970s through low-cost air travel, which facilitated the move towards globalisation. However, this has accelerated exponentially in the 21^st^ Century as technology and digitisation has led to the emergence of a virtual environment, which can be further exploited. This allows offenders to organise and commit serious crime, across international borders, without being physically present.

This specific study provided evidence from two independent sources of data to show that the threat posed by offenders who travel to, and commit offences in, the UK has increased. The FNS sample showed a significant proportion of all those arrested originate from across the globe. As the most common offence was violence related, rather than online fraud, drug trafficking, or money laundering, the sample do not immediately match the general perception of serious organised crime (SOC) offences. However, a range of factors could be relevant here. During the three-month snapshot there is a cohort of persistent offenders as 33% of the FNS sample had been arrested more than once and 8% had been arrested on at least three occasions. Further, it is impossible to say whether any of the violent offences were associated with the enforcement of organised crime activity (Kirby and Snow, 2016), or whether the thefts were synonymous with SOC (i.e. systematic shoplifting in high value jewellers). Conversely, perhaps it reflects the fact that it is more difficult apprehending offenders who engage in SOC, particularly if they remain remote from the crime scene (Curtis and Oxburgh, 2023). A study by Kirby et al. (2016) found only 0.2% of offenders, convicted in the UK over a three-year period, could be associated with a SOC offence. Such conjecture can only be established with further research using more detailed data. What can be stated is that the mobility associated with these arrests does create increased work for law enforcement when checking their identity, status and background.

Whilst it cannot be known what proportion of the FNS sample is associated with SOC it is clear there is a “significant foreign national involvement in serious and organised crime” (NCA, 2023: 5; Police Foundation, 2017). Previous studies in the UK have estimated this is between 13% (Kirby et al., 2016) and 22% (HM Government, 2013). It was this aspect of mobility that focused the attention of practitioners in the second element of the study. Indeed, practitioners reference to poly-criminality was symptomatic of the way offenders embraced new market opportunities, technology, and systems to increase their mobility and avoid detection. For example, the preponderance of fraud offences during the covid pandemic as well as the exploitation of illicit medication markets, using the dark web and community lockers, showed offender adaptability.

The practitioners were also consistent in arguing that whilst offenders have grasped the opportunities generated by mobility, there is concern that law enforcement agencies have not responded to the threat with sufficient speed. The NIM, which emerged in 2000, provided a step change in acknowledging the importance of mobility, categorising policing into levels 1 (local), 2 (regional) and 3 (national / international) (ACPO, 2007). However, this change did not provide a complete solution, and further structural changes were introduced to assist in monitoring mobile offenders and sharing information. As an example, the UK saw the introduction of the GLAA in 2005, ROCUs in 2009, the Border Force in 2012, and the NCA in 2013. Structural modifications have also been introduced in other countries. A US example encompasses fusion centres, which were formed to ensure the sharing of multi-agency information following the 9/11 terrorist attack. These increased from nine centres in 2003 to 80 centres in 2023 (Department of Homeland Security, 2023). However, like many other structural solutions they have been criticised in terms of their effectiveness and efficiency (Taylor and Russell, 2012).

The reason why structural interventions like this fail to deliver a complete solution has been illustrated in this study. Practitioners argued the proliferation of OCGs and the speed in which they change or adapt their behaviour is at a greater pace than government agencies can respond. Law enforcement intelligence systems are siloed, fragmented and often criticised. For practitioners to understand the full picture, numerous pieces of individual information must be identified as relevant and passed to the appropriate organisation. This means that data management and information sharing is an increasingly important component within law enforcement practice. Unfortunately, as offenders have become increasingly mobile the volume of data has grown exponentially and the ability to access it has become more complex as practitioners have to navigate the different values, legislation, and protocols attached to international jurisdictions.

There have been a few attempts to solve this dilemma. One common solution, proposed by a UK Royal Commission as far back as 1962 and reiterated as recently as 2022 (The Police Foundation, 2022), is to amalgamate police agencies into larger entities (i.e. national or regional organisations). This would potentially allow them to work from single intelligence systems. Whilst conceptually straightforward, and regularly suggested (Jones et al., 2024), attempts to implement this approach in the UK have proved futile as local jurisdictions defended their position, making amalgamation impossible without significant legislation and funding changes. Newer pragmatic approaches are therefore required if information sharing is to be improved.

The current practice of sharing information within law enforcement environments has been explained through problems associated with technology, legislation, and organisational culture (Phythian and Kirby, 2022). Further, law enforcement systems are heavily human dependent, relying on practitioners to identify what is important and what should be shared (Phythian et al., 2024). Human reliant systems are expensive to run as the practitioner must check numerous sources. They also suffer from intelligence and/or implementation failure due to human bias or a lack of knowledge or training. However, the introduction of the UK based PND in 2011 provides grounds for optimism. This national database is in fact an amalgam of 230+ separate crime, custody, domestic abuse, and child abuse records which have been assembled from various UK law enforcement agencies. The system allows an analyst to input a single search term in relation to people (i.e., offenders), objects (i.e., stolen property), locations (i.e., address) and events (i.e., a crime report), which is interrogated across numerous databases simultaneously. Advances in technology have made the feasibility of sharing intelligence from different jurisdictions, across a common platform, even easier to do.

Perhaps this development can act as a 21^st^ Century catalyst for a new paradigm in intelligence-led policing. The private sector has acknowledged the ability to exploit new business opportunities is often predicated on effective data management. The same issues are inherent when tackling SOC – it relies on the ability to capture, analyse and disseminate relevant information in more efficient ways. However, this needs a paradigm shift - from systems that predominantly rely on human discretion and effort, to machine reliant systems, and the use of artificial intelligence, that sift information more quickly to identify criminal activity. This can save human time by searching quicker at scale, as well as providing insight by highlighting patterns not immediately apparent to the human eye (Harris et al., 2023). By connecting disparate databases and using evidence-based algorithms it is possible to conduct an initial wash of millions of data points, to focus practitioners on those people or events that may be worth further investigation. For example, recidivist crime gang members can be cross referenced with transport and locations to identify concentrations of unusual behaviour which suggest criminality.

Whilst the ability to introduce such systems is technically possible, the PND project has indicated the difficulties associated with this type of innovation. Although sophisticated automated systems are more likely to push the right information to the right people at the right time, they are costly to design and maintain, as well as taking away some of the control and discretion enjoyed by practitioners. Further, implementation requires government and law enforcement leaders to recognise the shifting balance between local and international level criminality. Most importantly, it requires practitioners to overcome cultural issues, such as trust, which regularly make the sharing of information problematic. Nonetheless, to keep pace with criminal behaviour, law enforcement requires transformative thinking and action.
